# Case Report: Gutter endoleak embolization in a patient with bilateral common iliac artery aneurysms treated with sandwich parallel grafts

**DOI:** 10.3389/fsurg.2024.1518417

**Published:** 2024-12-19

**Authors:** Sangho Lee, Hyeonju Kim, Seung Huh, Hyung-Kee Kim

**Affiliations:** ^1^ Division of Vascular and Endovascular Surgery, Department of Surgery, Kyungpook National University Hospital, Daegu, Republic of Korea; ^2^ School of Medicine, Kyungpook National University, Daegu, Republic of Korea; ^3^ Division of Vascular and Endovascular Surgery, Department of Surgery, Kyungpook National University Chilgok Hospital, Daegu, Republic of Korea

**Keywords:** common iliac artery, aneurysm, parallel grafts, gutter, endoleak

## Abstract

**Background:**

Current guidelines recommend preserving at least one of the bilateral pelvic flows in patients with aortoiliac aneurysms. The sandwich parallel graft, using commercially available devices, provides a viable option for patients who fall outside the instructions for use of iliac branch devices. However, gutter endoleak remains a significant challenge.

**Case report:**

A 78-year-old woman with an incidentally discovered small AAA and bilateral CIAAs experienced aneurysmal growth to 42 mm for the abdominal aortic aneurysm (AAA) and 41 mm and 33 mm for the right and left common iliac artery aneurysms (CIAAs), respectively. Morphologic assessment of the aortoiliac aneurysm revealed the nearly 90-degree angles of the bilateral iliac bifurcations and the tortuous path of the internal iliac arteries. The iliac branch device was considered relatively contraindicated and ineligible; therefore, to preserve pelvic blood flow, the decision was made to employ the sandwich parallel graft technique in the left iliac arteries. Following the placement of the sandwich graft within the iliac limb, standard procedures were carried out. However, completion angiography revealed a type IA endoleak and a significant gutter endoleak in the left CIAA extending to the AAA sac. Despite the extension of overlapping zone, the gutter endoleak persisted after a 10 min waiting period. Therefore, we then advanced an 014 wire and a microcatheter to selectively access the gutter endoleak and performed embolization using multiple detachable coils. The final angiography showed complete resolution of the gutter endoleak and computed tomography angiography 1-month postoperatively confirmed the absence of any endoleaks.

**Conclusion:**

This case report highlights that targeted embolization is a feasible and effective treatment for significant gutter endoleak following the sandwich parallel graft technique.

## 1 Introduction

Abdominal aortic aneurysm (AAA) frequently coexists with common iliac artery aneurysms (CIAAs), with approximately 16%–43% of AAAs accompanied by at least unilateral CIAA, and bilateral CIAAs present in about 11%–12% of cases (1).

Current guidelines advocate for the preservation of at least one bilateral pelvic flow in patients with aortoiliac aneurysms (2, 3). Treatment strategies include iliac branch devices (IBDs), flared limbs for cases without significant common iliac artery aneurysmal dilatation, sandwich parallel grafts, and hybrid procedures involving external-to-internal iliac artery bypass grafts (4). However, each technique presents its own set of advantages and limitations. The sandwich parallel graft, utilizing commercially available devices, is particularly recommended for patients who fall outside the instructions for the use of IBDs. Nonetheless, type III endoleak due to gutter formation is a well-documented complication and remains a significant concern.

In this report, we present a case of a small AAA with bilateral CIAAs treated using sandwich parallel grafts in the left iliac arteries, coupled with right internal iliac artery embolization. Despite a significant gutter endoleak post-procedure, it was successfully addressed with gutter embolization. We selected this case to explore the considerations involved in selecting parallel graft diameter in the sandwich technique and to outline management strategies for gutter endoleak.

## 2 Case description

**Figure 1:**
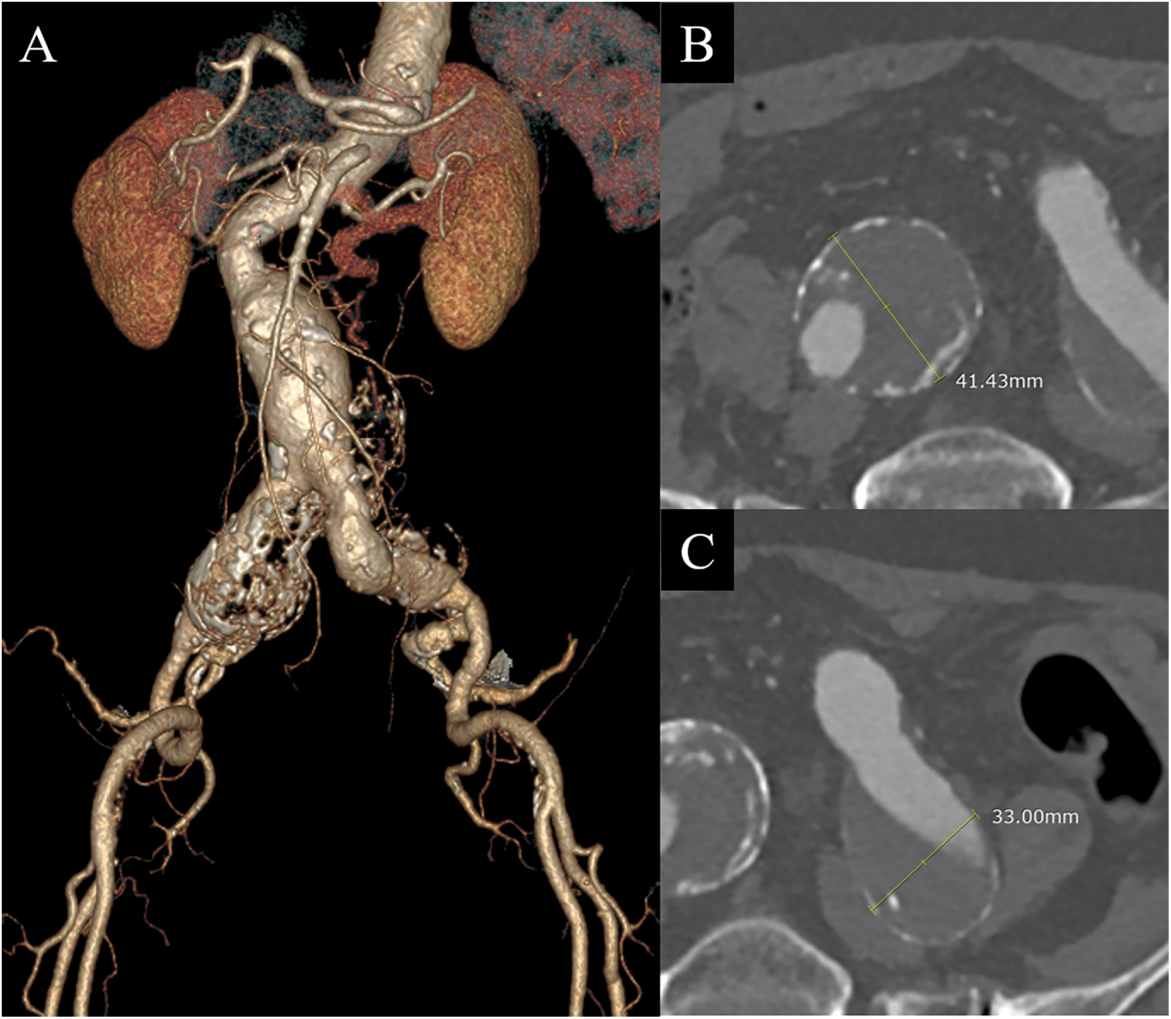
Initial computed tomography angiography (CTA) before endovascular aneurysm repair revealed a small abdominal aortic aneurysm and bilateral common iliac artery aneurysms (CIAAs). **(A)** 3-dimensional reconstruction image. **(B)** Axial image of the right CIAA demonstrating a 41 mm diameter aneurysm. **(C)** Axial image of the left CIAA showing a 33 mm aneurysm.

A 78-year-old woman initially presented with leg pain, during which a lumbar magnetic resonance imaging scan revealed an incidentally discovered small AAA and bilateral CIAAs 4 years ago. At that time, subsequent computed tomography angiography (CTA) confirmed the measurements: the AAA was 33 mm, the right CIAA was 32 mm, and the left CIAA was 27 mm. Over the subsequent 4 years, the aneurysms showed progressive growth to 42 mm for the AAA and 41 mm and 33 mm for the right and left CIAAs, respectively (Figure 1). Her medical history was notable for hypertension, coronary artery disease, and hyperlipidemia, for which she was receiving regular medications. Additionally, she had undergone a total laparoscopic hysterectomy with bilateral salpingo-oophorectomy for early-stage cervical cancer 1 year prior to this presentation. After thorough discussions regarding the diameter of the right CIAA, the associated risk of rupture, and the advantages and disadvantages of various treatment options, she opted to proceed with endovascular aneurysm repair (EVAR).

Morphologic assessment of the aortoiliac aneurysm revealed severe aortic neck angulation of 77 degrees; however, the aortic neck length was relatively long at 40.8 mm, the neck diameter measured 18 mm, and there was no evidence of a conical neck or reverse taper, making it possible for EVAR. The CIAA lengths measured 77 mm on the right and 78 mm on the left. Notably, the angles of the bilateral iliac bifurcations were wide, with 90 degrees on the right and 85 degrees on the left. Due to the nearly 90-degree angles of the bilateral iliac bifurcations and the tortuous path of the internal iliac arteries (IIAs), the IBD was considered relatively contraindicated and ineligible (5). Consequently, to preserve pelvic blood flow, the decision was made to employ the sandwich parallel graft technique in the left iliac arteries using two self-expanding Covera™ Plus stent grafts (Becton Dickinson & Co.) within the limb extension graft.

**Figure 2:**
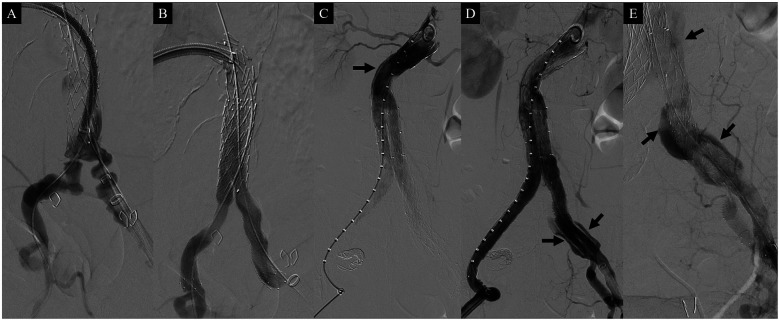
Intraoperative angiography during the sandwich parallel graft procedure. **(A)** Angiography showing the deployed InCraft iliac limb (Cordis Corporation) in the left common iliac artery and a wire passing through the internal iliac artery. **(B)** Post-deployment angiography of the parallel Covera™ Plus stent grafts (Becton Dickinson & Co.) within the InCraft limb graft, demonstrating well-patent parallel grafts. **(C)** Completion angiography revealing a type Ia endoleak (arrow). **(D)** Angiography also showing a gutter endoleak (arrows). **(E)** Gutter endoleak communicating with the sac of the abdominal aortic aneurysm (arrows).

The length of the left CIA and the angle of the aortic bifurcation were deemed sufficient for the up-and-over deployment of a stent graft to the left IIA, similar to the IBD procedure. As a result, we opted not to use the brachial approach for this parallel graft procedure. Initially, a 13 × 80 mm InCraft iliac limb (Cordis Corporation) was deployed in the left CIA. A through-and-through wire was then created within the deployed limb graft, extending between the right and left common femoral arteries (CFAs) via a separate puncture of the left CFA. An up-and-over 12F Ansel sheath (Cook) was advanced along this through-and-through wire to the distal part of the limb graft, and an additional wire from the right CFA was crossed into the distal left IIA (Figure 2A). Sandwich Covera™ stents, 100 mm in length, were then positioned within the iliac arteries [9 mm diameter for the IIA and 10 mm diameter for the external iliac artery (EIA)] (Figure 2B). Following the placement of the sandwich graft within the iliac limb, standard procedures were carried out, including right IIA coil embolization, main body deployment, overlapping stent graft placement, and limb extension to the right EIA. The main body diameter was 22 mm, resulting in an oversizing of 22.2% relative to the 18 mm neck diameter.

**Figure 3:**
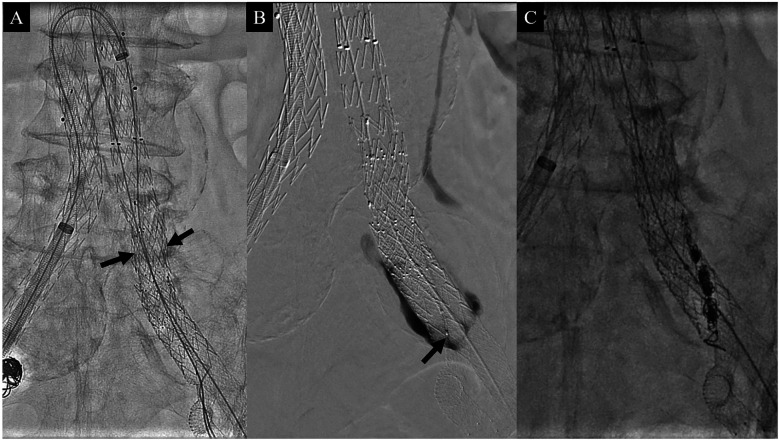
Intraoperative photographs. **(A)** After up-and-over placement of a 12F Ansel sheath from the right common femoral artery, two additional parallel stent grafts, each 60 mm in length, were placed proximal to the previous parallel grafts (arrows). **(B)** Despite proximal extension of the parallel grafts, the gutter endoleak persisted. Angiography following gutter selection with a microcatheter (arrow) showing the gutter endoleak. **(C)** Photograph showing multiple coils after gutter embolization.

**Figure 4:**
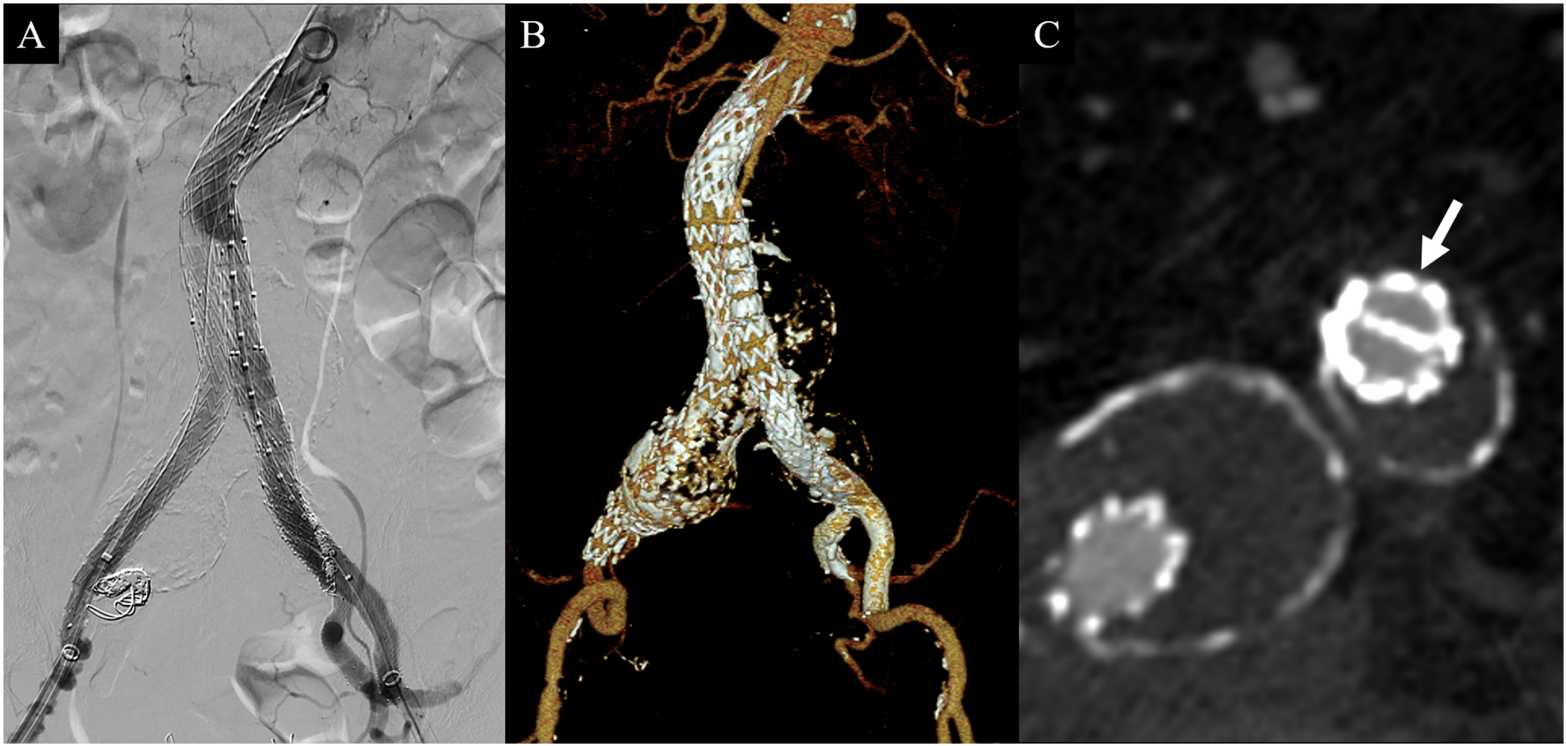
Final angiography during the operation and computed tomography angiography (CTA) 1-month postoperatively. **(A)** Final angiography after gutter embolization and Palmaz stent placement revealing no type Ia or gutter endoleaks. **(B)** 3-dimensional reconstruction image of follow-up CTA demonstrating well-patent parallel stent grafts without gutter endoleak. **(C)** Proximal portion of the parallel stent graft showing a double D configuration (arrow) without endoleak.

Completion angiography, however, revealed a type IA endoleak and a significant gutter endoleak in the left CIA extending to the AAA sac (Figures 2C–E). The type IA endoleak occurred at the greater curvature of the aortic neck, likely due to insufficient oversizing of the stent graft relative to the neck diameter. To manage the gutter endoleak, we initially suspected that the overlapping zone between the limb graft and the sandwich parallel grafts might be too short. Consequently, additional parallel grafts with diameters of 10 mm and 9 mm and lengths of 60 mm were deployed to extend the overlapping zone (Figure 3A). Despite this, the gutter endoleak persisted after a 10 min waiting period. We then advanced an angled microcatheter (Trailblazer™, Medtronic) proximally to the parallel grafts, as it allowed for angle adjustments to facilitate the entry of a 0.014 inch wire (Command ES™, Abbott) into the gutter. The soft tip of the wire was manipulated through several attempts until its position and folding outside the stent grafts were confirmed, indicating successful entry into the gutter. The Trailblazer microcatheter was subsequently advanced over the wire to access the gutter endoleak, and angiography was performed to verify the location (Figure 3B). Embolization was then carried out using four Concerto™ detachable coils (Medtronic): three coils with a diameter of 4 × 100 mm and one coil with a diameter of 5 × 200 mm, successfully sealing the gutter endoleak (Figure 3C). To address the proximal type IA endoleak, we deployed a balloon-expandable Palmaz stent (Cordis Corporation). Following the placement of the Palmaz stent, final angiography showed complete resolution of the gutter endoleak (Figure 4A). The patient was discharged on postoperative day 4 without any complications. CTA 1-month postoperatively confirmed the absence of any endoleaks and demonstrated patent sandwich parallel grafts with a double-D configuration (Figures 4B,C). Follow-up CTA at 6 months postoperatively revealed no abnormalities, including sac size increase or endoleaks.

This study, serving as a care report, adhered to the CARE (CAse REport) guidelines. This study was approved by the institutional review board; informed consent was obtained from the patient (approval number: KNUCH 2024-10-038).

## 3 Discussion

In the endovascular treatment of CIAAs concurrent with AAA, unilateral CIAA is typically managed by embolizing the ipsilateral IIA and extending the stent graft to the EIA. However, this approach becomes problematic in cases of bilateral CIAAs, as occluding both IIAs can lead to complications such as buttock claudication and sexual dysfunction, with reported incidences ranging from 13% to 55% (6). Although severe complications like gluteal necrosis, spinal cord ischemia, or colonic infarction are rare, their impact on the patient's quality of life can be profound (5, 7). To mitigate these risks, it is generally recommended to preserve at least one IIA whenever possible (2, 3).

Several fully endovascular options are available for bilateral CIAAs. One such option is the “bell-bottom” or flared iliac stent graft, which is suitable for small-sized CIAAs (8). However, this method is limited to CIAAs with a diameter of up to 28 mm and carries the risk of early and late type 1b endoleaks (9). Another method for preserving the IIA in cases of bilateral CIAAs is the use of an IBD (10). However, IBD is unsuitable for CIAs with a distal diameter smaller than 18 mm, a length shorter than 40 mm, a narrow aortic bifurcation, no distal landing zone in the IIA, or a wide iliac bifurcation (10, 11). Review papers indicate that the currently available Cook and Gore IBDs meet anatomical criteria in only 35% of cases combined (12). Even when expanding the criteria for IBD usage, only 58% of aortoiliac aneurysm patients fall within these parameters (5). In East Asian populations, the proportion of patients meeting the IFU criteria for IBDs is even lower, with approximately 17% in Japan and only about 9.8% in China (13, 14).

To address the anatomical limitations of IBD, the sandwich technique can be employed without the need for additional equipment beyond a covered stent (15). When planning the sandwich technique, it is essential to select the appropriate sizes for the stent grafts, ensuring an overlap of parallel grafts within the mother stent graft of more than 5 cm to prevent gutter endoleak (10). The size of the stent grafts used in the sandwich technique is typically determined based on the surgeon's preference, taking into account the circumference or area of the stent.

In Pang's 2019 study, the size of the stent grafts was determined based on circumference. The diameter of parallel stent grafts was calculated using the formula *c* = 2*πr* = 2*R* + 2*πR*/2 (where *c* = circumference, *r* = radius of the parallel stent graft, *R* = radius of the mother stent graft), yielding a ratio of *r*/*R* ≈ 4/5. In this study, gutter endoleaks occurred in 4 out of 13 patients; of these, 3 resolved spontaneously, and 1 required re-intervention with coil embolization (16). However, this method did not account for the thickness of the stent graft itself. To address this issue, Koussayer et al. (17) introduced a new formula that incorporates these factors: *D* = [0.68 × (*D*1 + *D*2)] + 2 (where *D* = diameter of the mother stent graft, *D*1 = diameter of the stent graft to the IIA, *D*2 = diameter of the stent graft to the EIA). In their study, gutter endoleaks were identified in 3 out of 10 sandwich parallel grafts, all of which resolved spontaneously. Unlike the circumference-based formula, DeRubertis et al. (18) determined stent graft size based on area, selecting two covered stents of similar diameter and radial force such that their combined cross-sectional area (*πr*^2^ + *πr*^2^) slightly exceeded the area of the iliac limb into which they were implanted. In their study, gutter endoleak occurred in 2 out of 21 patients, and all resolved spontaneously (18).

The “eye of the tiger” technique, a modification of the sandwich method, was developed to address challenges arising from varying stent graft sizes, which can lead to compression of smaller stents and gutter endoleak (19). This approach typically involves using a self-expanding stent graft for the EIA and balloon-expandable stent grafts for the IIA to minimize gutter space. By simplifying the sizing process, this technique ensures that the balloon-expandable IIA stent effectively seals the gutters in the standard sandwich technique. However, due to the limited clinical observation period for this modified method, it should be employed with caution (20).

In our case, we utilized an area-based calculation method. The cross-sectional area of the 13 mm iliac limb initially deployed in the left CIA was 132.67 mm^2^. Consequently, we selected a 10 mm Covera™ stent graft for the EIA (area: 78.5 mm^2^) and a 9 mm stent graft for the IIA (area: 63.59 mm^2^). The combined area of the parallel stent grafts was 142.09 mm^2^, slightly exceeding the area of the iliac limb. Although the procedure itself proceeded smoothly, a gutter endoleak was detected despite additional overlapping of the stent grafts, which was eventually managed with gutter embolization.

The Covera™ Plus covered stent used in our case is versatile and has been employed in procedures such as chimney EVAR and fenestrated EVAR (21). However, its application in the sandwich technique for pelvic flow preservation is less common (21). We chose the Covera™ Plus stent because it was the only self-expanding stent graft available in South Korea and allowed us to ensure symmetrical radial force by using stents of similar sizes from the same brand. Despite extending the overlapping region with additional stent grafts to address the gutter endoleak, the endoleak persisted. Given the significant amount of endoleak observed on angiography, we targeted the gutter space with a microcatheter and performed embolization. Due to the limited research on the use of the Covera™ Plus stent in parallel stent graft techniques, further data collection and studies are warranted.

While most gutter endoleaks resolve naturally, some cases may require re-intervention (16, 18, 22, 23). When intraoperative angiography reveals a minimal gutter endoleak, spontaneous resolution can often be anticipated; however, clear and substantial endoleaks may necessitate intraoperative embolization or future re-interventions.

In summary, this case report demonstrates that targeted embolization is a feasible and effective treatment for significant gutter endoleak following the sandwich parallel graft technique in complex aortoiliac aneurysms. Although most gutter endoleaks resolve spontaneously, targeted intervention can be a valuable option in significant cases.

## Data Availability

The raw data supporting the conclusions of this article will be made available by the authors, without undue reservation.
